# First-Time Fathers' Experience of Support from Midwives in Maternity Clinics: An Interview Study

**DOI:** 10.1155/2018/9618036

**Published:** 2018-11-08

**Authors:** L. Huusko, S. Sjöberg, A. Ekström, E. Hertfelt Wahn, S. Thorstensson

**Affiliations:** ^1^Närhälsan Skövde Women's Health Clinic, Skövde, Sweden; ^2^Women's Health Clinic, Umeå, Sweden; ^3^School of Health and Education, University of Skövde, Skövde, Sweden; ^4^Department of Health Sciences, University West, Trollhättan, Sweden

## Abstract

**Background:**

Research shows that first-time fathers want to take part in preparation for birth and parenthood but they describe being excluded by health professionals.

**Aim:**

The aim of this study was to illustrate first-time fathers' experiences of support from midwives in maternity clinics as a step in the validation of “The Father Perceived-Professional-Support” (The FaPPS) scale.

**Methods:**

A qualitative content analysis with an inductive and deductive approach was used; seven first-time fathers were strategically selected and interviewed. In the inductive part the following open question was asked: “How did you perceive the support from the antenatal midwife/midwives?” In the deductive part, the fathers were asked to respond to the FaPPS scale, in order to receive their thoughts and understanding of the scale, inspired by the “Think-aloud” method.

**Findings:**

The inductive results showed two main categories:* Experience of not knowing what support they needed* and* Experience of being excluded*. The fathers found support from other fathers in parental education classes, but they lack time to discuss. Overall it seems as if the fathers answered both from their own perspective and from the mothers' perspective. This was not evident in the deductive results. The FaPPs scale should therefore include* professionals' ability to strengthen social support from other first-time fathers *and* professionals' ability to offer support to the mother. Conclusion and Clinical Implications.* The fathers experienced exclusion both by themselves and also by midwives. Midwives should offer both parents the opportunity to pose questions. It is important for expectant fathers that time for discussion is planned in parental education classes. The FaPPS scale is useful but needs further development. Parts of our result are in line with earlier research, for decades; therefore it is necessary to focus more on support for fathers.

## 1. Background

Fathers want to take part in preparations for parenthood and birth and to be able to support their spouse [1–5]. Women also expect support and participation from their partner [5–9] which create a sense of security for women [10, 11]. When fathers are supported to take part in pregnancy and childbirth they grow more supportive towards their spouse [1]. Health professionals should support both women and their partners in the transition towards birth and parenthood [12] which will increase the chance of a positive childbirth-experience [13]. However, first-time fathers describe lack of support during pregnancy [14, 15]. Fathers are mostly content with support from midwives [14, 16] but they want more support and guidance in their role as fathers-to-be [1, 5]. Feelings of helplessness, worry, and fear can be difficult to talk about and fathers need support from health professionals to handle these emotions [1, 17, 18]. Being able to take part in the care of their newborn baby is satisfactory but fathers experience a lack of information for them as fathers [14, 16, 19]. Fathers understand that most of the information is aimed towards the woman [14, 16] but they want more information directed towards their own needs and they want health professionals to involve them in discussions around birth [3, 15]. Professional support is then important for fathers but they lack this support much too often [20]. Fathers feel too often excluded from parental education, examinations, and health care visits, which can lead to feelings that they have no-one to turn to [14, 16]. Fathers also see women as the main parent [16] as do grand-parents and some midwives [20]. Important for fathers' participation in parenthood is women's attitudes and that fathers have the possibility for parental leave [16, 21].

First-time fathers are in need of professional support in their new role as a future parent but they describe a lack of this support. More knowledge is needed about how first-time fathers' experience support from midwives in maternity clinics. Therefore the aim of this study was to illustrate first-time fathers' experiences of support from midwives in maternity clinics as a step in the validation of “The Father Perceived-Professional-Support” (The FaPPS) scale.

## 2. Material and Method

For this study, a qualitative design with both an inductive and deductive approach was selected [22] inspired by the “Think-aloud” method [23]. Individual interviews were used for data collection to capture the direct voices of the fathers, an important factor when aiming to measure abstract phenomena [24, 25]. For the inductive part, the interviews were performed using an open question. For the deductive part, interviews continued with questions based on the FaPPS scale items, described in the FaPPS section. The aim of using this method was to highlight the participants' perceptions [23] of their experiences of professional support in relation to the FaPPS scale items. The data was analysed using both inductive and deductive qualitative content analysis [26].

### 2.1. Setting

The study was performed in two antenatal maternity clinics in the southwest of Sweden. The municipalities where these clinics are situated had 51 000 and 18 000 inhabitants each and approximately 470 first-time mothers in total, per year. During pregnancy there were 10–11 individual visits with the midwife and first-time parents were also invited to take part in parental education groups for 3-4 meetings.

### 2.2. Participants

A purposive sampling strategy [27] was adopted, aiming for a variation in age, and education level. This variation enabled us to capture a wider variety of experience of professional support at the maternity clinic. First-time fathers who were included, had taken part in meetings at the clinic and their spouses had uncomplicated pregnancies. The seven participating first-time fathers varied in age from 21 to 42 years, and in education level from ground school to university exams. They all had participated in parental education and clinical meetings with the midwife. Two of the fathers had experienced that their child had been born at the time of the interview.

### 2.3. The FaPPS Scale

The FaPPS (Father-Perceived-Professional-Support) scale consists of a question about how fathers perceived the professional support from health care professionals, such as midwives, followed by eight statements: sensitive/not at all sensitive; understanding/not at all understanding; supportive/not at all supportive; had plenty of time/had very little time; gave enough information about breastfeeding/did not give enough information about breastfeeding; were calm/were stressed; provided good preparations for the role as a parent/provided no preparations for the role as a parent and gave good information about the baby's needs/gave no information about the baby's needs. The fathers were asked to grade their perceptions on a 1-7-grade scale that was sometimes reversed in order to avoid routine-like responses [27]. This scale is similar to the MoPPs scale about Swedish women's experiences of midwifery support during pregnancy [28].

### 2.4. Data Collection Procedure

Midwives at the maternity clinic approached the fathers. When they had accepted to participate they were contacted by the authors (LH or SS). The interviews were performed at the maternity clinic or at the fathers' home by choice of the individual father. The interview started with the inductive part, using an open question: “*How did you perceive the support that the midwife/midwives offered you in meetings at the maternity clinic?*” The question was posed to get the fathers own words of the professional support they had received from the midwives. During the interviews, the fathers were encouraged to reflect on their experiences and probing questions were used, such as:* Please, explain more, please explain how you experienced/perceived it? *The probing questions were used to encourage the interviewees to describe how they perceived the professional support. Thereafter, in the deductive part, the fathers were asked to write their answers in the FaPPS scale, in order to receive their thoughts and understanding of the scale. The fathers were also asked to reflect on each item and explain why they answered the way they did, inspired by the “Think-aloud” method [23]. The interviews were all recorded and lasted between 20 and 50 minutes.

### 2.5. Data Analysis

For the open questions, an inductive qualitative content analysis was used [26] to explore the direct experience of the fathers. In short, the transcripts from the interviews were read through several times, discussed, compared, and validated. Words and sentences relevant to the research questions were identified as meaning units, which were then condensed and coded, grouped under subcategories and organized into categories.

Regarding the deductive part, answers to the FaPPS scale items, a deductive qualitative content analysis was used [26]. The fathers' answers for each item was read through and analysed to understand their thoughts for each item, and the meaning of each item was identified. When fathers gave a lower (4 or less) or higher (5 or more) answer on the FaPPS scale items, their individual descriptions of these FaPPS scale items were analysed and compiled (Table 1). This procedure allowed for comparison in order to understand what inference could be drawn [25] from the different items of the FaPPS scale. This also allowed for a deeper understanding about the scale items and contributed to validation of the FaPPS scale.

### 2.6. Ethical Considerations

This study was conducted in accordance with the ethical guidelines of the Helsinki Declaration [29]. Approval from the Ethical Review Board of Gothenburg (EPN) was obtained (no. 405 09) before any data collection began. The head of the maternity clinics was given information and gave us access to undertake this study. The participating first-time fathers were given written and oral information about the study, as well as information explaining that their participation was voluntary and that they could withdraw from the study at any time without having to provide a reason and without the care of their spouse being affected. All data was handled with confidentiality.

## 3. Findings

### 3.1. Findings from the Open Questions: The Inductive Part

Analyses from the open questions resulted in two main categories “Experience of not knowing what support they needed” and “Experience of being excluded”. The first main category included two subcategories. An overview of the findings is presented in Table 2.

### 3.2. Experience of Not Knowing What Support They Needed

The fathers described that it was hard for them to know what kind of support they needed since they were becoming fathers for the first time. They did not have any specific questions to pose to the midwives since they did not know what to ask for. The fathers also described that they felt confident and prepared for their father-role and they had a longing to become parents. The fathers described that their parenthood would come automatically when their baby was born and it was not possible to prepare completely beforehand. Having experience with children, even if not their own, contributed to this sense of confidence.

*“It will probably be shocking whatever you do”…“You have to live and learn”*. (Interview 7)

*Informative Support from Midwives.* The fathers experienced that midwives had answered questions that they asked. They had been able to pose the question they wanted to ask without being questioned.

*“Our midwife always answered all strange questions that we asked”.* (Interview 1)

The fathers experienced the climate at the maternity clinics as open and warm. The midwives were described as calm and providing relevant information. When the fathers were worried the midwives could calm them. The midwives were also described as dedicated and professional. The fathers felt they had been involved during pregnancy. However, information and support from the midwives was not spontaneous and the fathers felt they had to show an interest themselves and ask questions to get involved during clinical visits.

*“I got the support I asked for”*. (Interview 7)

The fathers described that they benefited from the information that midwives offered the women and they described a relation to the midwife that grew more positive with time. Initially, they did not have the same confidence for midwives as they did now. The fathers described that they would not have contacted the midwife themselves between scheduled visits. It would have been strange for them since the mother would be better to explain certain questions around the pregnancy. The fathers described a lack of support from midwives directed towards them as fathers. The difference was that the mother was pregnant and was giving birth but they were both becoming parents. The fathers lacked someone to discuss in private regarding pregnancy and how life will change, as they become parents. The fathers found answers on their own use of the Internet and also turned to friends, family and colleges that had experiences from parenting. 

*Support from Other First-Time Fathers in Parental Education Groups.* Fathers described they got support during parental education group meetings. They experienced a need for information about pregnancy and birth and they got this information at these meetings. The fathers were positive towards these meetings but they found it difficult to absorb all the information that was presented by the midwives. They perceived the information as relevant and it was positive that the midwives shared their experience and knowledge both in theory and in practice. The fathers described they had gained an understanding of how the women could experience their pregnancies, and the fathers were also more understanding of what they themselves were going through.

*“Glad to meet those who have been there, the others also would be mothers and fathers for the first time, it feels like you have more to discuss and talk about”*. (Interview 7)

The fathers also experienced social support through parental education groups. They met other parents that were in the same situation and could discuss with them. What the fathers lacked during these group meetings was more time for dialogue and discussion, and above all dialogue with other fathers. They thought it could be a good idea to have separate groups for fathers and mothers since they felt that fathers had other needs than mothers. These group meetings could also be a forum for questions that were hard to share with their own spouse or other close relatives. It would be more natural to discuss this with others who were in the same situation. The fathers felt less lonely when they heard other fathers describe their thoughts and reflections.

### 3.3. Experience of Being Excluded

The fathers described being left out both by their own choice or not by their own choice. They described how they were supposed to support and help the women during birth, but they were not supposed to be involved during pregnancy or the first months of the children's lives. The fathers described understanding their role during childbirth as offering practical support. They therefore excluded themselves during pregnancy and considered this as positive on one hand, on the other hand there were feelings of resentment, that this was not accurate. The fathers wanted the midwives to actively involve them more so they could feel as equal parents when the baby was born.

The fathers described it as important that they were present during maternity visits. They participated to gain knowledge about the baby but mostly as support for the women. The fathers wanted guidance on how to act and behave during pregnancy and birth.

*“Was the question aimed directly to support me? I could not answer that actually”*. (Interview 1)

The fathers did not see themselves as having the lead role during pregnancy but rather as the person that should secure that the woman was safe. The fathers said that they stood beside the woman that was, and should be, the centre of attention.

They felt they had no influence over the pregnancy and they could not understand what it was like to be pregnant. The fathers did not see themselves as equal parents in the first months of the baby's life. They meant that they could not meet the needs of the baby in the same way as mothers could, the baby could not survive without the mother. The fathers described that they could contribute with more practical things such as cocking and change nappies and in those ways facilitate things for the women. The fathers wanted to participate but they excluded themselves believing that this was best for the baby. The fathers also excluded themselves from the language they used. They spoke about their experiences in third person or in we, in questions aiming towards the fathers they also described mothers' experiences.

The fathers got concrete advice about how to act during birth and this was described as positive. They thought that this advice would help them support the women during birth. The fathers, however, lacked support that aimed directly to them. They described being welcomed by the midwives but they still felt invisible during visits.

*“It is as it is, you get a bit left out, it is probably automatically so as a guy actually, I think so”.* (Interview 6)

They had not been met unfriendly but no questions were aimed directly at them and this made the fathers feel not involved but excluded.

### 3.4. Findings Deductive Analysis

An overview of the findings of the FaPPS scale items is shown in Table 1. An overall description of the meaning of each item is followed by a description of how assessing high (5 or more) or low (4 or less) for each item was described (Table 1).

### 3.5. Comparing Inductive and Deductive Results

To gain a deeper understand of the FaPPS scale items and to validate the scale a comparative analysis of inductive and deductive results were performed. Overall inductive and deductive results were quite similar. However, fathers described in the inductive results that they felt excluded by midwives that did not meet their needs as an expectant parent. The fathers described being excluded both by their own choice and by the midwives, but this was not shown in the deductive results. In the inductive result, fathers described that they did not know what to expect since this was their first child and they felt it was impossible to prepare for their parental role or the child's needs, irrespective of how much information the midwives offered. This was also shown in the deductive results but more as satisfaction even with little or lack of information. Overall it seems as if the fathers answered both from their own perspective and from the mothers' perspective. It seems important that support towards the woman was included as an item in the FaPPs scale. The fathers found support from other first-time fathers in parental education classes, but they lacked time to discuss. This was not evident in the deductive results and the FaPPs scale should include professionals' ability to strengthen social support. Some of the words in the FaPPs scale were difficult to understand for some father and that the scale items sometimes were revised caused confusion.

## 4. Discussion

The result from our study showed that first-time fathers did not know what support they needed since this was their first child. The fathers found support when meeting other first-time fathers in parental education classes and they also found support when midwives offered relevant information. Health professionals should support both women and their partners in the transition towards birth and parenthood [12], which will increase the chance of a positive childbirth-experience [13]. Professional support, shall not include only informative support, it shall also include emotional and practical support, when needed [21]. The fathers excluded themselves by focusing on the mother, but they also felt excluded by midwives who did not meet their needs as expectant parents. The fathers found the informative support from midwives as valuable but the lack of support they described was in line with earlier research from Deave and colleagues [14, 20] who shows that fathers find professional support is primarily offered to mothers. However, fathers in our study also described that midwives answered their questions. Being able to pose questions is earlier described as a form of support by Bäckström et al. [2]. First-time fathers in our study also described both excluding themselves and being excluded from support. Fathers excluded themselves during pregnancy since they thought the mother should be in focus. On the other hand, fathers found this wrong since they were also expectant parents and wanted to be equal parents when the baby was born. Earlier research shows that fathers find it important that professional support from midwives towards fathers should not be at the expense of mothers [1–3]. Fathers in our study did not see themselves at the centre of attention during pregnancy but rather as the person providing for safety and security for the mother. During the interviews the fathers talked about themselves in third person, using the words “we” or “one” rather than “I”, which further strengthens the message of exclusion.

The fathers described that they thought they could not meet the babies' needs in the same way as the mothers could in the first months so they placed themselves beside the mothers. Earlier research describe the importance of midwives actively meeting fathers as parents-to-be and confirming their ability to meet the needs of their baby [16, 18]. The fathers felt welcomed by the midwives but at the same time invincible during meetings. They did not feel included when no questions were posed towards them. Earlier research describes that fathers do not see themselves as main parents [16] they stand beside and watch [14]. The question is if it is natural for fathers to stand beside or if midwives contribute to their feelings of exclusion? Our result also showed that the first-time fathers felt prepared for their parenting role since they felt it was not possible to prepare and it would become automatic once the baby was born. However, earlier research describes the opposite, that fathers feel unprepared in their parenting role once the baby is born [14]. This implies the importance for midwives actively including fathers when talking about preparations for birth and parenting.

Fathers in our study turned to friends and family for support during pregnancy, which is in line with earlier research. Fathers experience social support in parental education classes where they could discuss with other expectant parents and they felt less alone listening to other fathers. They also describe gaining more understanding about pregnancy and what both mothers and they themselves go through [2, 14]. Earlier research also describes the opposite that fathers feel excluded during parental education classes [14]. What fathers in our study lacked was time to discuss with other expectant fathers since they had different needs and thoughts than mothers. Professional support from midwives should also aim to strengthen social support [30, 31]. This implies the importance for midwives to plan for enough discussion time during parental education classes in order to strengthen social support for first-time fathers.

Our deductive result from the FaPPs scale items showed that the first-time fathers saw themselves as excluded and they blamed themselves when midwives showed a lack of sensitivity or understanding. The fathers meant that the midwives were focused on the mothers and the fathers did not initiate contact themselves. However, our result also showed that fathers felt included when the midwives had eye contact with both parents and/or posed questions directly to the father. Eye contact has been shown to instil security in patients, offering confirmation to them [32]. First-time fathers in our study found it important that they were allowed to pose any questions they needed. This is in line with earlier research showing that fathers' feel supported when they are able to pose any questions to the midwife [2]. The fathers in our study described it as negative when midwives did not listen or when their ideas were dismissed. They also found it negative when personal values of midwives were exposed in meetings. This is also in line with earlier research that professional support should be sensitive to individual needs [18].

The fathers in our study described that there was little information about the babies' needs and preparation for parenting and breastfeeding. The fathers felt that this information was not directed towards them but at the same time, they found it being sufficient, because it was directed to the mothers. Earlier research shows that parents feel that fathers do not need information about breastfeeding [20]. However, earlier research also shows that fathers' support is important for breastfeeding to succeed [33]. This highlights the importance of including the fathers in information about breastfeeding and the baby's needs.

Using qualitative content analysis, this study investigated first-time father's experiences and reflections of professional support during pregnancy as a step in the validation of the FaPPS scale. Individual interviews were chosen as data collection method; interviews were able to catch the father's narratives, which provided information about meaningful values, experiences, and reflections [34]. Throughout the study, different steps were considered to enhance the trustworthiness of the study [35]. The study is limited by its small sample size, but the context and the participants are described as clearly as possible to facilitate the transferability of the results [34]. Further, using purposive sampling, contributed to variation in age, and education and how many midwife visits they attended, which also is strengthening for the trustworthiness of the study [27, 36]. The study was pilot tested, we did not need to change the interview guide, and therefore we included the two pilot interviews in the results. Using both an inductive and deductive approach was useful in understanding first-time fathers' experiences of support from midwives in maternity clinics and contributed to a deeper understanding of the FaPPS scale items. This will be helpful in further development of the FaPPs Scale. A challenge is to make fathers actually answer from their own perspective only and not, as in this study, both from their own and from the mothers' perspective.

## 5. Conclusion and Clinical Implications

The findings of our study showed that fathers experienced exclusion by themselves which was evident in both the inductive and deductive results. However, feeling excluded by midwives was not evident in the deductive results and this needs to be addressed in the further development of the FaPPs scale. Professional support towards the woman was important for first-time fathers and this will need to be included in the FaPPs scale as well as professionals ability to strengthen social support for fathers.

Our result pointed out the importance of midwives having eye contact with both parents and actively posing question to both parents as well as offering both parents the opportunity to pose questions. It is important for first-time fathers that time for discussion is planned in parental education classes. Midwives should strive to include fathers in information about the baby's needs and breastfeeding since this is important for them as becoming parents. Parts of our result are in line with earlier research, for decades; therefore it is necessary to focus more of support for fathers.

## Figures and Tables

**Table 1:** Findings from fathers experience of professional support in relation to the FaPPS scale items.

**Item **	**Meaning of item**	**Answering lower**	**Answering higher**	**Range**
**Sensitive - Not at all sensitive **	Midwives ability to be sensitive toward fathers' needs. Fathers mean they are partly responsible for less sensitivity since they themselves focus on the women.	Midwives do not listen and do not take the ideas of the couple seriously	Midwives take time to answer questions of importance for the couple	4-7
**Understanding – not at all understanding **	Midwives understanding depend on fathers initiative or if they express feelings such as worry	The fathers themselves did not initiate dialogue and therefore the midwives did not offer any understanding.	The fathers got understanding from the midwives if they were worried.	4-7
**Supportive – not at all supportive **	Being supportive was having eye contact or pose questions to both parents and when midwives were supportive of the woman.	If the relationship with the midwives didn't work or if the midwives personal values were revealed during visits it was experienced as less supportive.	Fathers experienced midwives as supportive when they supported the women. Parental education was experienced as supportive.	4-7
**Hade plenty of time – had very little time **	Fathers could ask the midwives about anything they needed to ask.	Not relevant	There were no time pressure and the midwives were calm.	5-7
**Information about breastfeeding – Gave not enough information about breastfeeding **	Breastfeeding was the mothers' role and responsibility. General information and advice when or if breastfeeding does not work was needed.	No information about breastfeeding directly toward fathers.	There was no need for information about breastfeeding, the fathers knew what they needed to know.	3-7
**Were calm – were stressed**	Overall midwives were described as calm.	Not relevant	The environment was calm	5-7
**Gave good preparation for the parenting role – gave no preparation for the parenting role **	Fathers saw their role mainly to facilitate for the mother and baby. Fathers had gained information about their parenting role from family, friends and from the internet.	Pregnancy in focus and not their parenting role.	Enough information from midwives since it was not possible to prepare before the baby was born.	3-7
**Gave good information about the needs of the baby - gave no information about the needs of the baby **	Common sense was basic and the fathers would learn in time.	Some information about the baby's needs from midwives	Information from midwives was sufficient.	3-7

**Table 2:** Overview of findings from the open questions: the inductive part.

**Main category**	**Sub-categories**
Experience of not knowing what support they needed	(i) Informative support from midwives
	(ii) Support from other first-time-fathers in parental education groups

Experience of being excluded	

## Data Availability

The data have not been made available because of ethical reasons due to the area of the study being rather small and there is a small possibility that participant could be recognized if transcripts were available.
